# Prognosis of hypoglycemia episode in cirrhotic patients during hospitalization

**DOI:** 10.1186/s12876-021-01895-2

**Published:** 2021-08-09

**Authors:** Tsung-Hsing Hung, Chih-Wei Tseng, Chih-Chun Tsai, Hsing-Feng Lee

**Affiliations:** 1 Division of Gastroenterology, Department of Medicine, Dalin Tzu Chi Hospital, Buddhist Tzu Chi Medical Foundation, No. 2, Minsheng Rd., Dalin Township, Chiayi County 62247 Taiwan; 2 School of Medicine, Tzu Chi University, Hualien, Taiwan; 3 Department of Mathematics, Tamkang University, Tamsui, Taiwan

**Keywords:** Hypoglycemia, Cirrhosis, Mortality

## Abstract

**Background:**

Studies have shown that hyperglycemia in cirrhotic patients increases mortality. However, no population-based study has evaluated the influence of hypoglycemia upon hospital admission on death in these patients. The aim of this study was to assess the effect of hypoglycemia at admission on the mortality of patients with liver cirrhosis.

**Methods:**

The Taiwan National Health Insurance Database was searched, and 636 cirrhotic patients without baseline diabetes mellitus who presented with hypoglycemia upon hospitalized from 2010 to 2013 were included in the study. A one-to-four propensity score matching was performed to select a comparison group based on age, sex and comorbidities.

**Results:**

The overall 30-day mortality rate was 30.2% in the hypoglycemia group and 7.4% in the non-hypoglycemia group (*P* < 0.001). After Cox regression modeling adjusting for age, sex and comorbid disorders, cirrhotic patients with hypoglycemia had a hazard ratio (HR) of 30-day mortality of 4.96 (95% confidence interval [CI] 4.05–6.08, *P* < 0.001) as compared to the non-hypoglycemia group. In subgroup analysis, the cirrhotic patients with hypoglycemia and hepatocellular carcinoma (HCC) had a 30-day mortality HR of 6.11 (95% confidence interval [CI] 4.40–8.49, *P* < 0.001) compared to those with neither hypoglycemia nor HCC.

**Conclusions:**

Hypoglycemia is a very important prognostic factor in the 30-day mortality of cirrhotic patients, especially in those with underlying HCC.

**Supplementary Information:**

The online version contains supplementary material available at 10.1186/s12876-021-01895-2.

## Introduction

Liver is a metabolic organ that plays an important role in glucose metabolism. It maintains and regulates the blood sugar mainly through the glycogenolysis and gluconeogenesis. Liver dysfunction is well known to correlate with poor blood glucose regulation [1]. The presence of the disturbance of liver metabolism, or liver cell damage may decrease the stability of the liver in regulating the blood glucose level. Ascites, variceal bleeding, hepatic encephalopathy are mainly complications in patients with liver cirrhosis. It is worthy to know the prognosis in these patients with the hypoglycemia. Hypoglycemia is not uncommon in cirrhotic patients. In one report, hypoglycemia was noted in 50% patients with liver cirrhosis with bacetermia [2]. In one study, the survival analysis showed a significantly lower estimated survival for patients with hypoglycemia (36 days) than for patients with normal blood sugar (54 days) [3]. These findings may indicate the important role of hypoglycemia in patients with liver cirrhosis.

In addition, patients with liver cirrhosis are also prone to have hepatocellular carcinoma (HCC) [4]. Paraneoplastic syndrome may occur in patients with HCC who present with hypoglycemia [5]. Because both of the liver cirrhosis and HCC are possible factors correlated with hypoglycemia, it is worthy to realize the effect of the hypoglycemia on the mortality of these patients. Although a few studies have evaluated the role of blood glucose in the mortality of cirrhotic patients [3, 6, 7], no population-based studies have yet evaluated the effect of hypoglycemia on short-term mortality in cirrhotic patients. Using the Taiwan National Health Insurance Research Database (NHIRD), we designed this population-based study to identify the role of hypoglycemia in the short-term mortality of cirrhotic patients. In subgroup analysis, we evaluated the role of hypoglycemia in cirrhotic patients with different liver-related complications.

## Materials and methods

### Database and ethical statement

The National Health Insurance Administration (NHIA) in Taiwan instituted a National Health Insurance program that covers more than 99% of the Taiwan population. In this program, all enrolled medical institutions must provide medical records to the NHIA for medical payment. The medical records were established as a database, the NHIRD. This database includes International Classification of Diseases, 9th Revision, Clinical Modification (ICD-9-CM) codes, medical procedures, number of days of hospitalization, medications and other related information on patients in Taiwan who have been hospitalized.

This study was approved by the Institutional Review Board of the Buddhist Dalin Tzu Chi Hospital (IRB B10403026). Because all of the data in the NHIRD is de-identified, the Institutional Review Board waived the requirement for informed patient consent and the need of informed consent for ethical approval.

### Study sample

The database was searched for patients discharged between January 1, 2010 and December 31, 2013 with a primary or secondary diagnosis of cirrhosis (ICD-9-CM codes 571.5 or 571.2). These ICD-9 codes have been used in past studies to identify patients with cirrhosis in Taiwan. A total of 146,632 cirrhotic patients were screened. In clinical practice, an important common cause of hypoglycemia is the overdose of medications related to treat diabetes mellitus. In order to avoid the effect of these medications, patients with underlying diabetes mellitus were excluded from the study. Hypoglycemia was defined by ICD-9-CM code 251.2 in the database. A total of 102,523 cirrhotic patients without baseline diabetes mellitus were included. Of these, patients were enrolled only if they had hypoglycemia noted during the hospital stay. If a patient had multiple hospitalizations for hypoglycemia during the study period, only the first episode was included in the analysis. Finally, a total of 636 cirrhotic patients with hypoglycemia were enrolled. We used one-to-four propensity score matching to match a control group (non-hypoglycemia group) to the study group by age, gender and liver-related complications (the presence of either hepatic encephalopathy, variceal bleeding or ascites). We collected for analysis the confounding factors of age, gender, hepatic encephalopathy (ICD-9-CM code 572.2), alcoholism (ICD-9-CM codes 291, 303, 305.00—305.03, 571.0—571.3), HCC (ICD-9-CM code 155.0), ascites (ICD-9-CM code 789.5 or procedure code 54.91), variceal bleeding (ICD-9-CM codes 456.0, 456.20), renal function impairment (ICD-9-CM codes 584, 585, 586, 572.4, or codes for renal failure procedures), cachexia (ICD-9-CM codes 799.4), cancer (ICD-9-CM code 140-239), Charlson Comorbidity Index (CCI), socioeconomic status (SES), hypoalbuminemia, and bacterial infections. We used the presence of albumin supplementation to define the hypoalbuminemia in our study because the serum albumin level could not be identified from the database. In the cirrhotic patient with edema or ascites, albumin supplementation is covered by the health insurance program when the patient’s serum albumin level is lower than 2.5 g/dL [8]. A CCI ≧ 4 was defined as high [9]. The individuals in this study were classified into three groups: low SES, medium SES, and high SES. The monthly income lower than New Taiwan Dollar (NTD $ 20,000) (about US$ 556) was defined as low SES. The monthly income between NTD $20,001–40,000 (about US$ 556–1111) was defined as medium SES. The monthly income more than NTD $ 40,001 (about US$ 1111) was defined as high SES. The bacterial infections included were bacteremia, cellulitis, pneumonia, biliary tract infection, necrotizing fasciitis, empyema, brain abscess, urinary tract infection, septic arthritis, perianal abscess, liver abscess, bacterial meningitis and spontaneous bacterial peritonitis. The etiology of liver cirrhosis (such as hepatitis B or hepatitis C) could not be well obtained from the inpatient dataset we applied because of the coding limitation from this dataset. This is the reason the hepatitis B or C were not analyzed in this study.

Cirrhotic patients often also present with HCC (ICD-9-CM code 155.0). In subgroup analysis, we calculated the hazard ratios (HRs) of hypoglycemia in the short-term mortality among these patients with concurrent HCC.

### Statistical analyses

We used the chi square test to compare categorical variables, and Student’s t test to compare continuous variables. Propensity score was performed to match analysis including age, gender and underlying comorbidities to minimize potential confounding effects. The Cox regression model was used to identify the risk factors associated with mortality. The survival analysis was analyzed by the Kaplan–Meier method with the log‑rank test for univariate analysis and the proportional hazards Cox regression model for multivariate analysis. Statistical significance was defined *P* < 0.05. All Data were analyzed by the SPSS statistical package for Windows version 22.0 (IBM Corp., Armonk, NY).

## Results

**Fig. 1:**
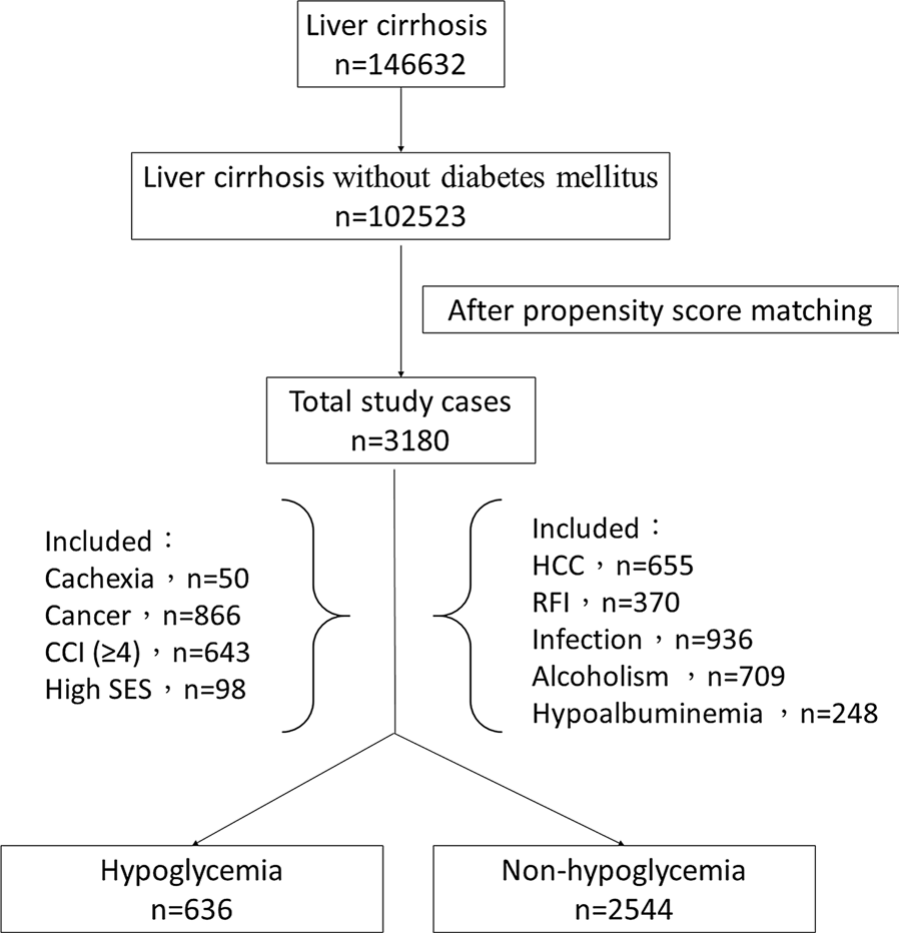
The flowchart of this study. *HCC* hepatocellular carcinoma, *RFI* renal function impairment, *CCI* Charlson Comorbidity Index, *High SES* High socioeconomic status

**Fig. 2:**
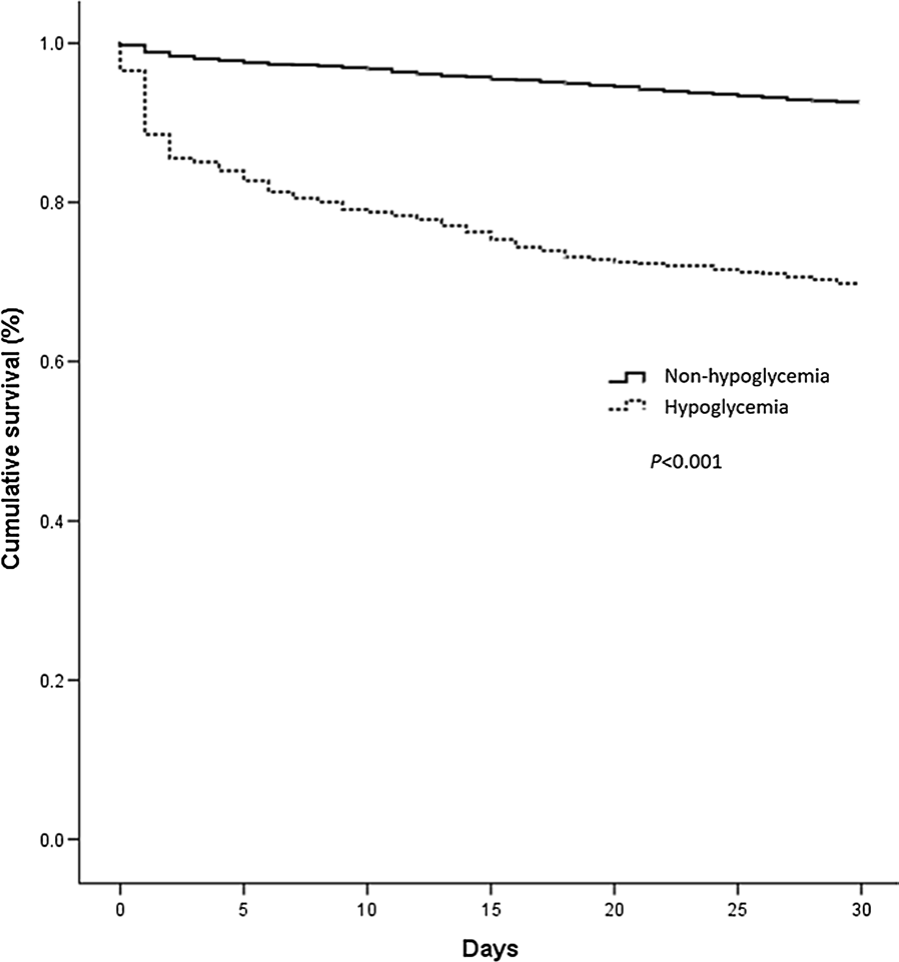
Kaplan–Meier survival analysis for survival of cirrhotic patients with and without hypoglycemia during the 30‑day follow up

The database was searched for patients discharged between January 1, 2010 and December 31, 2013 with the diagnosis of liver cirrhosis. After review of the database and application of the inclusion and exclusion criteria, 636 patients with cirrhosis and hypoglycemia were included in the study as the hypoglycemia group. With 1:4 propensity score matching, 2544 cirrhotic patients without hypoglycemia were included as the non-hypoglycemia group. Of the 636 cirrhotic patients with hypoglycemia, the mean age was 60.61 ± 15.5 years and 485 (76.3%) were males. Of the 2544 cirrhotic patients without hypoglycemia, the mean age was 60.22 ± 15.3 years and 1979 (77.8%) were males. Table 1 shows the demographic characteristics of the two groups. Because of the propensity score matching, factors such as cancer, alcoholism, renal function impairment, complication conditions, cachexia, SES, CCI, hypoalbuminemia, bacterial infections, the cirrhosis-related complications, gender and age were not significantly different between the two groups. The overall 30-day mortality was 30.2% for the hypoglycemia group and 7.4% for the non-hypoglycemia group (*P* < 0.001). After Cox regression modeling adjusting for age, sex and other comorbid disorders, the HR for 30-day mortality of the hypoglycemia group was 4.96 (95% CI 4.05–6.08, *P* < 0.001) times that of the non-hypoglycemia group. Other risk factors for 30-day mortality of cirrhotic patients included one cirrhotic-related complication (HR, 2.04; 95% CI 1.63–2.56; *P* < 0.001), RFI (HR, 2.59; 95% CI 1.99–3.38; *P* < 0.001), bacterial infections (HR, 1.67; 95% CI 1.35–2.07; *P* < 0.001), cachexia (HR, 2.14; 95% CI 1.28–3.57; *P* = 0.004), cancer (HR, 3.06; 95% CI 2.38–3.94; *P* < 0.001) and hypoalbuminemia (HR, 2.35; 95% CI 1.81–3.05; *P* < 0.001). Other factors, including male (HR, 1.23; 95% CI 0.95–1.60; *P* = 0.116), age (HR, 1.00; 95% CI 1.00–1.01; *P* = 0.330), two or three cirrhotic-related complications (HR, 1.50; 95% CI 0.88–2.58; *P* = 0.138), alcoholism (HR, 0.93; 95% CI 0.68–1.28; *P* = 0.649), CCI (≥ 4) (HR, 0.88; 95% CI 0.68–1.13; *P* = 0.309), and SES do not reveal significant difference in this study. The statistically significant prognostic factors were summarized in Table 2. The flowchart for this study were shown in Fig. 1. The cumulative survival plots for the patients with and without hypoglycemia were shown in Fig. 2. Cirrhotic patients with hypoglycemia had a significantly lower cumulative survival rate than those without hypoglycemia (*P* < 0.001).

**Table 1:** Demographic characteristics of the hypoglycemia groups

	Hypoglycemia (+) (n = 636)	Hypoglycemia (−) (n = 2544)	*P* value
Male	485 (76.3)	1979 (77.8)	0.408
Age, y	60.61 ± 15.5	60.22 ± 15.3	0.569
Complication conditions			0.668
No complication	480 (75.5)	1939 (76.2)	
1 complication	141 (22.2)	533 (21.0)	
2 or 3 complications	15 (2.4)	72 (2.8)	
HCC	143 (22.5)	512 (20.1)	0.188
RFI	77 (12.1)	293 (11.5)	0.678
Infection	185 (29.1)	751 (29.5)	0.831
Alcoholism	139 (21.9)	570 (22.4)	0.766
Cachexia	10 (1.6)	40 (1.6)	1.000
Cancer	184 (28.9)	682 (26.8)	0.282
Socioeconomic status			0.936
Low	409 (64.3)	1644 (64.6)	
Medium	206 (32.4)	823 (32.4)	
High	21 (3.3)	77 (3.0)	
CCI (≥ 4)	134 (21.1)	509 (20.0)	0.551
Hypoabluminemia	54 (8.5)	194 (7.6)	0.467

Age presented as mean ± standard deviation; other data as number (percentage)

*HCC* hepatocellular carcinoma, *CCI* Charlson Comorbidity Index, *RFI* renal function impairment

**Table 2:** Adjusted hazard ratios of risk factor for 30-day mortality of cirrhotic patients

Variable	Hazard ratio	95% Confidence interval	*P* value
Male	1.23	0.95–1.60	0.116
Age, y	1.00	1.00–1.01	0.330
Complication conditions			
No complication			< 0.001
1 complication	2.04	1.63–2.56	< 0.001
2 or 3 complications	1.50	0.88–2.58	0.138
RFI	2.59	1.99–3.38	< 0.001
Infection	1.67	1.35–2.07	< 0.001
Alcoholism	0.93	0.68–1.28	0.649
Cachexia	2.14	1.28–3.57	0.004
Cancer	3.06	2.38–3.94	< 0.001
Socioeconomic status			
Low			0.210
Medium	1.04	0.84–1.29	0.722
High	0.58	0.30–1.10	0.093
CCI (≥ 4)	0.88	0.68–1.13	0.309
Hypoglycemia	4.96	4.05–6.08	< 0.001
Hypoalbuminemia	2.35	1.81–3.05	< 0.001

*RFI* renal function impairment, *CCI* Charlson Comorbidity Index

To evaluate the role of hypoglycemia in cirrhotic patients with HCC, subgroup analysis were performed. In cirrhotic patients with underlying HCC, the HR for 30-day mortality of those with hypoglycemia was 6.11 (95% CI 4.40–8.49, *P* < 0.001) times that of those without hypoglycemia. In cirrhotic patients without underlying HCC, the HR for 30-day mortality for those with hypoglycemia was 4.51 [95% CI 3.47–5.86, *P* < 0.001] times that of those without hypoglycemia. For confirming the result, we also performed the Cox regression as the main analysis without propensity score matching. The results were listed as Additional file 1: Appendix 1. The hypoglycemia is still an important prognostic factor in the 30-day mortality of cirrhotic patients.

## Discussion

In this present study, we demonstrated that cirrhotic patients who had a hypoglycemic episode during hospital admission had a higher 30-day mortality rate than those without hypoglycemia. In addition, the cirrhotic patients with concurrent HCC also had a higher 30-day mortality rate than those without HCC.

To our best knowledge, this is the first population-based study to discuss short-tern mortality in cirrhotic patients with a hypoglycemic episode during admission. Our present findings were similar to those of a previous study. Pfortmueller et al. showed that hypoglycemia is associated with increased mortality in cirrhotic patients with acute decompensation [3]. However, our study revealed that cirrhotic patients had higher short-term mortality regardless of the decompensated status.

In this present study, we also showed that cirrhotic patients with concurrent HCC had a higher mortality rate than those without HCC. Paraneoplastic syndromes are not uncommon in HCC patients. The incidence rate was reported to range from 4 to 27% [10]. Several studies have been shown that HCC patients with paraneoplastic syndromes, including hypoglycemia, have poor prognosis [5, 11, 12]. Therefore, not surprisingly, in our present study, cirrhotic patients with concurrent HCC had poor prognosis.

Some reasons might explain why cirrhotic patients with a hypoglycemic episode during admission are at higher risk of short-term mortality. First, hypoglycemia is a sign of malnutrition. Dozens of studies have shown that malnutrition contributes to increased mortality during admission [13–15]. Second, patients with severe infection with sepsis have a poor prognosis when hypoglycemia occurs at admission [16–18]. In one recent publication, Furukawa et al. found higher mortality in septic patients with hypoglycemia and hypoalbuminemia at admission [16]. Thus, these septic patients are in greater need of immediate intensive treatment during admission. In other words, according to our current findings, cirrhotic patients who had a hypoglycemic episode during admission were also those who needed intensive care. Third, hypoglycemia may be associated with adrenal insufficiency [19–21]. Liver cirrhosis has been reported as being to some degree an adrenocortical dysfunction [19, 22, 23]. Thus, cirrhotic patients with hypoglycemia have an inappropriately low response of the adrenal glands to stimulation and increased mortality. Lastly, severe liver fibrotic disease is associated with poor glucogensis [24]. In one review article, Olson et al. held that hypoglycemia may be as sign of acute liver failure with impaired glucogensis, and reflect the impending loss of the remaining compensatory mechanism [3, 24]. All of these factors may explain the poor prognosis in these cirrhotic patients with hypoglycemia.

There are some limitations in our present study. First, this dataset did not identify the etiology of liver cirrhosis. Nevertheless, the most important factor contributing to non-alcoholic liver cirrhosis in Taiwan is chronic viral infection, including hepatitis B or/and hepatitis C [25]. Therefore, adding the etiology of cirrhosis would not affect the results. Moreover, Child–Pugh scores and the Mayo Clinic Model for End-stage Liver Disease scores were also unavailable. In this dataset, it was not possible to use the ICD-9 codes to identify laboratory data such as concentrations of bilirubin or albumin or prothrombin time. However, we were able to track cirrhosis-related complications such hepatic encephalopathy, ascites and esophageal variceal bleeding, to represent cirrhotic patients with decompensated status. Second, because of the baseline difference between the study and comparison groups, we used propensity score matching before Cox regression. However, the unmatched group is not included in the analysis after the propensity score matching method used. This reduces the generalizability of the study results and makes the lack of robustness of our final analysis results. In order to confirm the results, we also performed the Cox regression as the main analysis without propensity score matching. The results were listed as Additional file 1: Appendix 1. The crude HR for 30-day of cirrhotic patients with hypoglycemia before and after propensity score matching method were 6.76 and 4.78 (*P* < 0.001) compared to patients without hypoglycemia. After Cox regression analysis, the adjusted HR for 30-day of cirrhotic patients with hypoglycemia before and after propensity score matching method were 5.45 and 4.96 (*P* < 0.001), respectively. The survival of cirrhotic patients with hypoglycemia were affected by many factors, such as underlying diseases, liver reserved, infections, …etc. In fact, we could not analyze all factors by Cox regression method, and these factors may skew the results before and after adjusting for confounders. However, the hypoglycemia is still an important prognostic factor in the 30-day mortality of cirrhotic patients regardless of the propensity score matching or Cox regression analysis. Finally, the hypoglycemia was defined by ICD-9 coding number and we could not validate that. This is the limitation of this database we used. However, we excluded the patients with underlying diabetes mellitus. In clinical practice, an important common cause of hypoglycemia is the overdose of medications related to treat diabetes mellitus. This may partially decrease the bias of this study.

In conclusion, this nationwide population-based study showed that hypoglycemia is a very important prognostic factor in the 30-day mortality of cirrhotic patients, especially in those with underlying HCC.

## Supplementary Information

**Additional file 1.** Appendix 1.

## Data Availability

The data that support the findings of this study are available from National Health Insurance Research Database (NHIRD) in Taiwan but restrictions apply to the availability of these data, which were used under license for the current study, and so are not publicly available. Data are however available from the authors upon reasonable request and with permission of NHIRD in Taiwan.
