# Supplementary material for: Prognosis of hypoglycemia episode in cirrhotic patients during hospitalization
Source: BMC Gastroenterol. 2021 Aug 9;21:319. doi: 10.1186/s12876-021-01895-2 (PMC8351368; doi:10.1186/s12876-021-01895-2)
Supplement: Supplementary file 1 — Additional file 1. Appendix 1. [file 12876_2021_1895_MOESM1_ESM.doc]

**Appendix 1**

**Table 1. Demographic characteristics of the overall cirrhotic patients before propensity score matching**

|  | Hypoglycemia (+)  (n=639) | Hypoglycemia (-)  (n=101884) | P value |
| --- | --- | --- | --- |
| Male | 488 (76.4) | 74325 (73.0) | 0.052 |
| Age, y | 60.57 ± 15.5 | 59.69 ± 15.4 | 0.151 |
| Complication conditions |  |  | 0.001 |
| No complication | 483 (75.6) | 81524 (80.0) |  |
| 1 complication | 141 (22.1) | 17010 (16.7) |  |
| 2 or 3 complications | 15 (2.3) | 3350 (3.3) |  |
| HCC | 143 (22.4) | 24633 (20.1) | 0.290 |
| RFI | 78 (12.2) | 5414 (5.3) | <0.001 |
| Infection | 186 (29.1) | 23731 (23.3) | 0.001 |
| Alcoholism | 141 (22.1) | 19890 (19.5) | 0.106 |
| Cachexia | 10 (1.6) | 147 (0.1) | <0.001 |
| Cancer | 184 (28.8) | 30985 (30.4) | 0.376 |
| Socioeconomic status |  |  | <0.001 |
| Low | 409 (64.3) | 57056 (56.4) |  |
| Medium | 206 (32.4) | 38400 (38.0) |  |
| High | 21 (3.3) | 5635 (5.6) |  |
| CCI (≥4) | 134 (21.0) | 16645 (16.3) | 0.002 |
| Hypoabluminemia | 54 (8.5) | 7378 (7.2) | 0.240 |

Age presented as mean ± standard deviation; other data as number (percentage).

Abbreviations: HCC, hepatocellular carcinoma; CCL, Charlson Comorbidity Index; RFI, renal function impairment.

**Table 2. Adjusted hazard ratios of risk factors for 30-day mortality of cirrhotic patients before propensity score matching**

| Variable | Hazard ratio | 95% Confidence Interval | P value |
| --- | --- | --- | --- |
| Male | 1.32 | 1.24 - 1.41 | <0.001 |
| Age, y | 1.02 | 1.02 - 1.02 | <0.001 |
| Complication conditions |  |  | <0.001 |
| No complication |  |  | <0.001 |
| 1 complication | 2.69 | 2.53 - 2.89 | <0.001 |
| 2 or 3 complications | 3.47 | 3.14 -3.84 | <0.001 |
| RFI | 3.14 | 2.92 - 3.37 | <0.001 |
| Infection | 1.97 | 1.87 - 2.09 | <0.001 |
| Alcoholism | 1.08 | 1.00 - 1.17 | 0.041 |
| Cachexia | 3.24 | 2.40 – 4.37 | <0.001 |
| Cancer | 2.09 | 1.97 – 2.23 | <0.001 |
| Socioeconomic status |  |  | 0.001 |
| Low |  |  | 0.210 |
| Medium | 0.98 | 0.93 - 1.03 | 0.445 |
| High | 0.75 | 0.65 – 0.87 | <0.001 |
| CCI (≥4) | 1.32 | 1.23 – 1.40 | <0.001 |
| Hypoglycemia | 5.45 | 4.71 – 6.31 | <0.001 |
| Hypoalbuminemia | 2.26 | 2.11 – 2.41 | <0.001 |

Abbreviations: RFI, renal function impairment; CCI, Charlson Comorbidity Index
